# Uveal Melanoma Biopsy: A Review

**DOI:** 10.3390/cancers11081075

**Published:** 2019-07-30

**Authors:** Luisa Frizziero, Edoardo Midena, Sara Trainiti, Davide Londei, Laura Bonaldi, Silvia Bini, Raffaele Parrozzani

**Affiliations:** 1 IRCCS-Istituto di Ricovero e Cura a Carattere Scientifico—Fondazione Bietti, 00198 Rome, Italy; 2 Department of Ophthalmology, University of Padova, 35128 Padova, Italy; 3 Immunology and Molecular Oncology Unit, Veneto Institute of Oncology, IOV-IRCCS – Istituto di Ricovero e Cura a Carattere Scientifico, 35128 Padova, Italy

**Keywords:** uveal melanoma, prognosis, biopsy, fine needle aspiration biopsy, metastases

## Abstract

Intraocular tumor diagnosis is based on clinical findings supported by additional imaging tools, such as ultrasound, optical coherence tomography and angiographic techniques, usually without the need for invasive procedures or tissue sampling. Despite improvements in the local treatment of uveal melanoma (UM), the prevention and treatment of the metastatic disease remain unsolved, and nearly 50% of patients develop liver metastasis. The current model suggests that tumor cells have already spread by the time of diagnosis, remaining dormant until there are favorable conditions. Tumor sampling procedures at the time of primary tumor diagnosis/treatment are therefore now commonly performed, usually not to confirm the diagnosis of UM, but to obtain a tissue sample for prognostication, to assess patient’s specific metastatic risk. Moreover, several studies are ongoing to identify genes specific to UM tumorigenesis, leading to several potential targeted therapeutic strategies. Genetic information can also influence the surveillance timing and metastatic screening type of patients affected by UM. In spite of the widespread use of biopsies in general surgical practice, in ophthalmic oncology the indications and contraindications for tumor biopsy continue to be under debate. The purpose of this review paper is to critically evaluate the role of uveal melanoma biopsy in ophthalmic oncology.

## 1. Introduction

**Figure 1:**
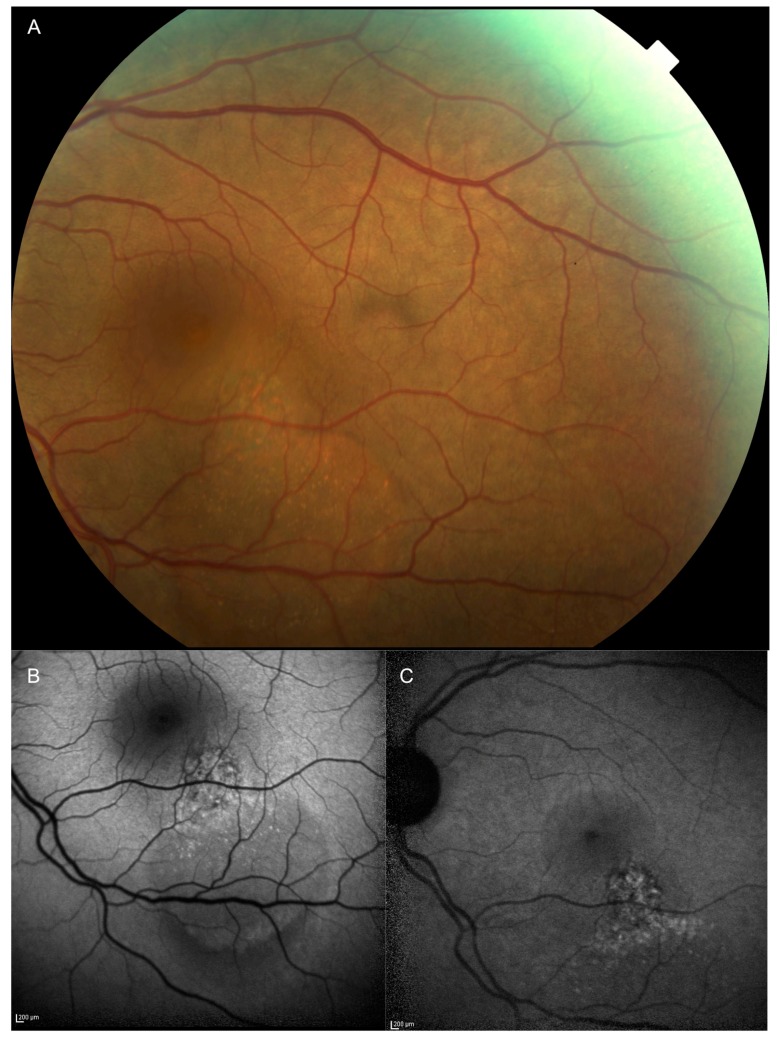
(**A**) Fundus photograph and (**B**) blue (**C**) and infrared autofluorescence of a case of a macular small uveal melanoma characterized by (**A**–**C**) diffuse orange pigment on its surface and (**B**,**C**) serous retinal detachment. The thickness of this lesion measured by spectral domain optical coherence tomography was 550 μm.

In clinical oncology, the treatment of malignant tumors requires the histologic confirmation of the initial diagnosis. For intraocular tumors, such as uveal melanoma (UM), the treatment decision is based on the clinical examination and ancillary testing, such as ultrasonography, fluorescein and indocyanine angiography, optical coherence tomography and auto-fluorescence [1]. Unfortunately, this is not proven for small uveal melanocytic lesions (thickness < 3 mm and a largest basal diameter < 10 mm [2] (Figure 1A–C).

The current management of small choroidal indeterminate pigmented lesions (encompassing atypical nevi as well as small melanomas) is either a periodical observation until growth or treatment, whereas clinical oncology practice often considers an earlier diagnosis (and treatment) as a first and mandatory step to improve patient survival [3]. Moreover, to biopsy an intraocular malignant tumor is still a controversy because of the theoretical risk of tumor dissemination due to the invasive procedure, and the small size and posterior location of the lesions increase the risk of insufficient sampling and potentially sight-threatening ocular complications [4,5].

**Figure 2:**
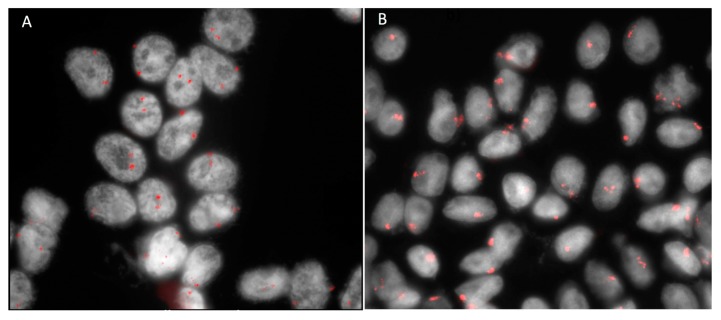
Fluorescence in situ hybridization (FISH) analysis with centromeric probe for chromosome 3 of tumor material obtained by fine needle aspiration biopsy in a case of uveal melanoma. (**A**) Normal cells with two red signals corresponding to two chromosomes 3. (**B**) Monosomy 3: cells with one red signal have lost one chromosome 3.

More recently, biopsy for genetic testing has become increasingly warranted due to the accuracy of cytogenetic prognostication and the landscape characterized by the emerging possibilities of personalized treatment regimens [6]. The genetic and molecular characterization of UM is more reliable, as a single modality, in prognosis prediction, compared to classical clinical and pathological features such as tumor dimensions, location and histological type [7,8,9] (Figure 2A,B).

Moreover, the continuous attempts for new molecular targets of systemic therapies in metastatic patients and the clinical trials enrolment based on the tumor molecular profile require a reliable genetic and molecular characterization, which can be obtained using different tumor biopsy techniques.

These considerations suggest the necessity of critically evaluating the role of UM biopsy in current clinical ocular oncology practice, better defining the main indications and contraindications of different sampling techniques.

## 2. Results

### 2.1. Intraocular Tumor Biopsy: Indications and Contraindications

**Figure 3:**
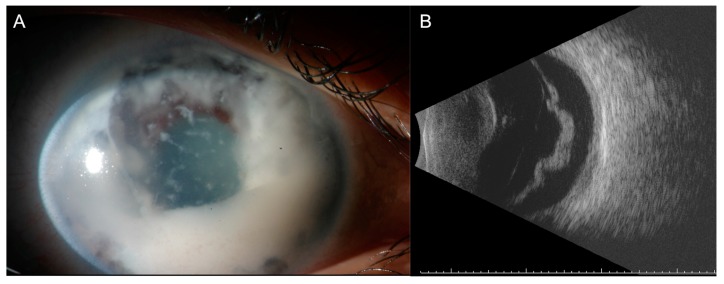
A case of post enucleation histologically proven diffuse retinoblastoma in an 8-year old child. Note (**A**) the anterior chamber invasion and (**B**) the increase retinal thickness of the detached retina in the B-Scan examination. (**B**) No calcifications are detectable by ultrasound.

The possible risk of inadequate sampling, iatrogenic ocular morbidity and the risk of extraocular tumor seeding have limited the diagnostic use of intraocular tumor biopsy for special cases with a significant diagnostic uncertainty [10]. The purpose of a diagnostic intraocular tumor biopsy is to confirm or rule-out the clinical suspicion of a malignancy. [10]. A main indication for diagnostic intraocular tumor biopsy remains diagnostic uncertainty, with conflicting results from non-invasive tests [10]. This assumes that a pathologic evaluation may result in a definitive diagnosis leading to the correct management. It may be also requested if the patient refuses treatment until the malignancy is confirmed. However, any procedure that might cause major morbidity and worsen the outcome should be avoided, excluding the use of biopsy as a shortcut to diagnosis [5,11,12]. It is especially important to avoid intraocular biopsy in children with suspect retinoblastoma, as well as in the case of an unusual clinical presentation (Figure 3A,B).

The risk of dissemination of these poorly cohesive tumors makes the suspicion of such a tumor a relatively strong contraindication [13]. Lesions that are supposed to be benign or patients with systemic disorders, such as tuberous sclerosis with hamartomas in multiple organs, are also generally not candidates for intraocular biopsy [14]. 

During the last two decades, the indication for intraocular biopsy has been completely revised due to the evolving possibilities of genetic prognostication. At present, cytogenetic testing may help assess the individual risk for metastasis, also changing the cascade screening and surveillance of the family members [5,10,11,12]. The use of UM biopsy for prognostication purposes is discussed in the paragraph “Biopsy for cytogenetic analysis”.

### 2.2. Intraocular Tumor Biopsy: Techniques

**Figure 4:**
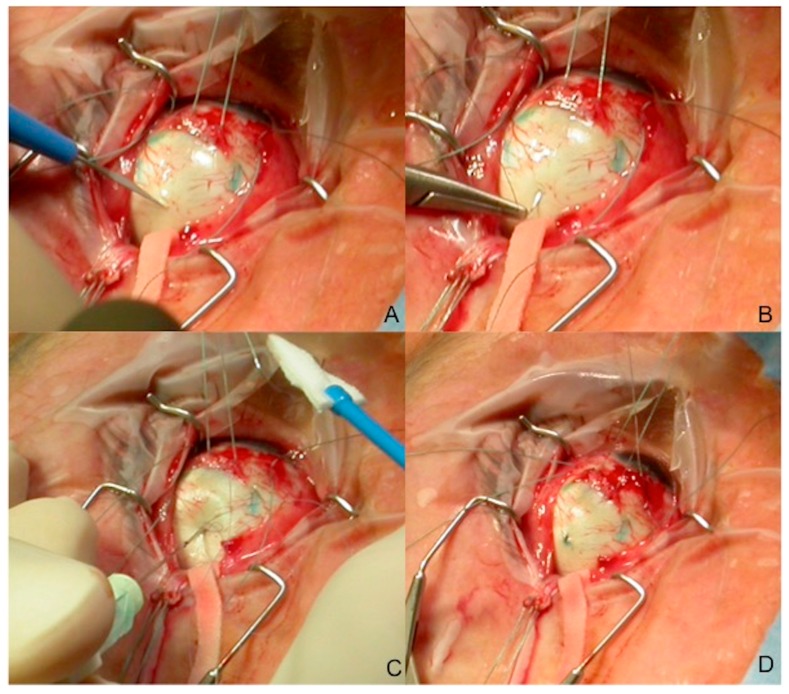
Fine needle aspiration biopsy sample: transscleral approach in a posterior uveal melanoma. Our standard fine needle aspiration biopsy (FNAB) procedure is performed using a 25 gauge (25 mm in length) spinal needle connected to a 10 cc syringe by a hollow tube. (**A**) The needle is inserted into the tumor trough a partial scleral incision (to avoid excessive pressure when penetrating the eye) (Figure 1). (**B**) The scleral suture (7.0 Polyglactin) is prepared before the needle insertion. (**C**) A double-pass or multiple-pass sampling is often performed through the same scleral access. (**D**) The scleral incision is then sutured and the radioactive plaque immediately placed over the tumor base.

There are several biopsy procedures for UM, which can be divided according to the location of the lesion [15]. For the anterior segment tumors, the described techniques include aqueous tap, iris fine needle aspiration biopsy (FNAB) or punch biopsy, surgical biopsy or biopsy using the vitrector. For the posterior segment tumors, the techniques include: FNAB performed transsclerally (Figure 4A–D) or transvitreally, vitrectomy-assisted approaches, punch biopsy, endoresection and transscleral resection [6,10]. All of these procedures are potentially diagnostic, with different complication rates and side effects.

### 2.3. Anterior Segment Tumors

The diagnosis of iris melanoma is carried out by clinical examination with slit-lamp biomicroscopy. For small tumors, anterior segment optical coherence tomography (AS-OCT) is useful. For large iris melanomas, ultrasound biomicroscopy (UBM) and AS-OCT assist in the visualization of the posterior tumor extent [16].

#### 2.3.1. Aqueous Tap

**Figure 5:**
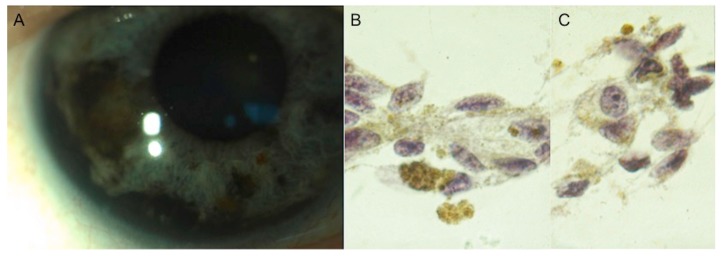
(**A**) A case of diffuse iris melanoma characterized by anterior chamber angle infiltration. (**B**,**C**) Aqueous tap of the same case confirming the diagnosis of spindle cell iris melanoma.

Aqueous tap may be an option to identify cellular infiltration in the anterior chamber. This technique should be considered as the first and less-invasive approach for selected iris lesions, with visible aqueous seeding, including iris melanomas, mainly the diffuse type (Figure 5A–C), and iris metastases [10,17,18].

Woog et al. reported a 46-year-old woman with a history of breast carcinoma and no known metastatic disease who presented with iridocyclitis and secondary glaucoma [18]. The cytological examination of the aqueous humor revealed adenocarcinoma. Char et al. reported a small series of histologically confirmed iris ring melanomas diagnosed by aqueous tap. The major limitation of this technique is that even in an optimal setting the specimens are paucicellular [17].

#### 2.3.2. Iris Fine Needle Aspiration Biopsy

**Figure 6:**
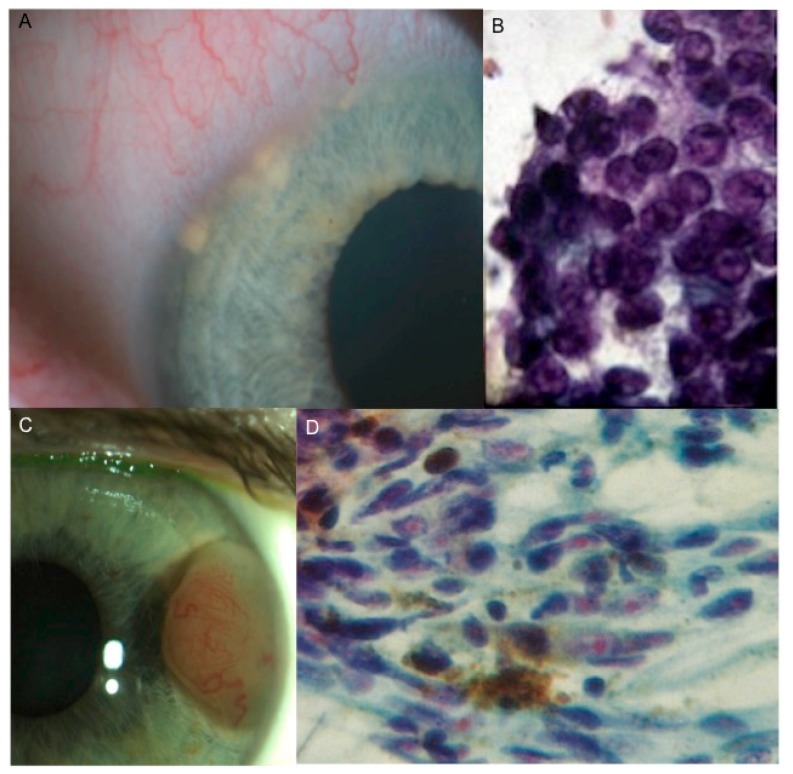
(**A**,**B**) A case of iris metastasis characterized by (**A**) multiple anterior chamber angle nodules in a patient previously treated by surgery and systemic chemotherapy because of a breast carcinoma (ductal type). (**B**) Intraocular fine needle aspiration biopsy of the same case confirming the diagnosis of iris metastasis from breast carcinoma. (**C**,**D**) A case of (**C**) iris partially amelanotic melanoma confirmed at cytology by (**D**) fine needle aspiration biopsy.

Iris FNAB involves proper instrumentation, the planning of the tumor approach, handling of the sampled cells, and preparation and interpretation of cytologic specimens [5,19]. It is important to realize that only a limited number of cells may be obtained through aspiration (Figure 6A–D).

Therefore, the surgical approach depends on location and tumor size. The standard technique for iris FNAB consists of a 1 mm limbal incision in the clear cornea and a viscoelastic material injection in the anterior chamber. Then, typically, a 25 gauge (G) sharp needle is placed through the corneal incision and aqueous, then into the iris tumor. The needle should be inserted through the cornea at an approximate 20–30° angle to the iris and, when inside the anterior chamber, the needle should be parallel to the iris [19]. The preferred entry site is approximately at 90° from the meridian of the tumor. A specific needling procedure (gently moving the needle back and forth into the lesion while always maintaining the vacuum) is recommended [5,19]. Scraping the tumor moves cells that are manually aspirated by an assistant through extension tubing. Though typically self-limited, hyphema is common. Shields et al. report 100 consecutive cases biopsied over a 24-year period and reported a diagnostic yield of 99% after obtaining one (74%), two (24%) or three (2%) specimens [19]. The authors concluded that FNAB appears as a safe and useful diagnostic technique, providing adequate cell sampling for cytological interpretation in nearly all cases. The major challenge occurs when the specimens are paucicellular and the decision must be based on a limited number of cells. The experience and skill of the cytopathologist are equally critical in all FNAB-based diagnoses [17,19].

#### 2.3.3. Iris Biopsy Using Vitreous Cutter

Bechrakis et al. reported 11 cases of iris tumor biopsy performed using a vitreous cutter through a 2-port clear cornea approach [20]. A 21-gauge infusion was inserted into the anterior chamber, and the intraocular pressure was elevated to 70 mmHg. A 20-gauge vitreous cutter was then inserted through the second limbal incision and placed on the tumor surface in such a way that its opening was occluded by tumor tissue. With a high aspiration setting (400 mmHg) and low cutting frequency (80/min), one single bite was obtained from the tumor surface. Tumor sampling was diagnostic in all cases. Although this technique appears to be safe and effective, Bechrakis et al. do not recommend its use in a routine clinical setting due to its costs [20]. Petousis et al. reported the results and complications of an aspiration cutter-assisted biopsy performed in fifty-five patients. This study suggests that small-incision, aspiration cutter-assisted biopsy seems to be a safe and effective procedure, without short- or long-term complications [21].

#### 2.3.4. Iris Surgical Biopsy

Surgical iridectomy may be useful in the case of unsuccessful FNAB sampling, since it yields adequate tissue for histopathology and immunohistochemical analysis. However, it involves a relatively large surgical corneal or scleral wound. [22]. Finger et al. described a minimally invasive method to create multifocal full-thickness surgical iridectomy biopsies [23]. Seven patients underwent a multifocal surgical iridectomy biopsy through a single 1.0-mm clear corneal incision. A 25-G inked trocar was used to create one visible corneal portal, through which the anterior chamber was filled with sodium hyaluronate 1%. Then, a 25-G aspiration cutter probe was introduced through the corneal incision, and the aspiration (600 mm Hg) cutting (300 cuts per minute) was used to create full- and partial-thickness surgical iridectomy biopsies at multiple locations. This technique provided adequate diagnostic material in most of the reported cases. The authors also described a rapid rehabilitation, and no significant complications [23]. In clinical practice, surgical iridectomy is mainly used as an excisional biopsy to remove all neoplastic tissue.

### 2.4. Posterior Segment Tumors

#### 2.4.1. Fine Needle Aspiration Biopsy

FNAB is widely used in the diagnosis of posterior segment tumors and can be performed both transvitreally and transsclerally [3]. The transvitreal (indirect) approach entails an anterior entry at pars plana, opposite to the tumor location and through the vitreous body and the retina into the choroidal tumor. In the transscleral (direct) approach, the sclera is punctured at the tumor base, and the tumor sample is obtained leaving the retina intact. The first procedure is most feasible when tumors are located posteriorly; the second one when they are located anteriorly or near the equator of the eye. These techniques are cheap and have been shown to carry little risk of ocular complications [24]. The main limitation is a low yield and consequential risk of insufficient sampling [25,26].

The basic equipment required for FNAB includes: a fine needle (25–30 G), and a 10 mL disposable syringe [5]. Most of the authors prefer a 25-gauge needle for both the transvitreal and trans-scleral approaches [10,11,27]. Other authors recommend a 30-G needle in transscleral procedures and a 27-G one for the transvitreal approach [3]. Shields et al. investigated the potential of the FNAB technique for small melanoma < 3 mm in thickness in 56 consecutive patients and found that a single-pass 27-G needle trans-pars plana transvitreal approach into the tumor apex was sufficient to obtain an adequate tumor sample in 97% of cases [3]. Transscleral FNAB is performed for ciliochoroidal and anterior choroidal tumors, usually just prior to the placement of the radiation implant. A partial thickness (about 80% scleral thickness) equilateral triangular scleral flap may be created at the entry site. The needle is inserted into the tumor through a 300 µm scleral incision one or more times. This specific procedure allows for safe sampling, avoiding retinal damage or intravitreous penetration by the needle. A double or triple pass sampling with the specific needling procedure is mandatory in all cases [5,11]. The scleral incision is immediately sutured after sampling. The use of a lamellar scleral flap in the transscleral technique was proposed to reduce concerns about the seeding of tumor cells in the subconjunctival space using a straight needle approach. In association with this, when FNAB is performed at the time of brachytherapy, a radioactive plaque is promptly placed over the tumor base after sampling, sterilizing the needle tract [11]. The use of the Essen forceps through a lamellar scleral flap later sealed by histoacryl glue, for the anteriorly placed UM, has also been proposed [28]. This technique seems to improve the specimen yield for the cytological and genetic analysis compared to the traditional transscleral FNAB technique, without any reported tumor recurrence [28]. For UM located posteriorly to the equator, a transvitreal approach may be more convenient, mainly because of a better tumor visualization. For transvitreal access, the needle should be bent 2–3 mm from its beveled tip to an angle of 60–90° relative to the shaft [29]. This allows entry into the neoplasm with the needle tip parallel to the retina, reducing the risk of posterior scleral perforation in small tumors [4]. The aspiration procedure may be repeated 2–3 times in order to obtain enough material, and the needle is withdrawn after cessation of the suction. Meticulous localization by indirect ophthalmoscopy is recommended. Alternatively, an operating microscope with a widefield viewing system may be used [26]. While the transscleral approach usually requires the tumor to be at least 3 mm in height, the transvitreal approach allows for direct transpupillary visualization during the procedure and sampling of small tumors [10]. However, the needle is usually inserted in the thickest part of the lesion, avoiding the retinal vessels. The procedure can be combined with the prior removal of the vitreous body (vitrectomy) to reduce the risk of persistent vitreous hemorrhage or vitreous traction on the iatrogenic retinal break at the tumor entry site, which usually self-seals [30]. The sclerotomies are sutured and cryotherapy may be performed [26]. Singh et al. investigated 71 transscleral FNAB and 64 transvitreal FNAB for UM. The yield was diagnostic in 92% of cases [27]. False-negative results were observed in 8%. The diagnostic yield was significantly correlated with the biopsy approach (Transscleral 96%, Transvitreal 86%; *p* = 0.029) and tumor size (basal diameter > 5.0 mm; height > 2.5 mm). Persistent hemorrhage (subretinal or vitreous hemorrhage) requiring surgical intervention (1%) and rhegmatogenous retinal detachment (1%) were rare. Endophthalmitis, hypotony, tumor recurrence, and episcleral seeding were not observed over the average follow-up of 37 months. The authors concluded that the procedure is safe, but that the possibility of an inadequate FNAB sample should be considered when counselling patients with small tumors [27]. Cohen et al. described similar results wherein the FNAB adequacy was correlated with the tumor height with yields of 40% in tumors < 2 mm in height and of 98% in tumors over 4 mm [31]. Augsburger et al., in a retrospective analysis, evaluated 302 cases of clinically diagnosed UM by FNAB from 1980 to 2006 [32]. In this case series, 260 (86.1%) samples yielded sufficient cells for cytopathologic classification. However, FNAB for a cytopathologic diagnosis remains significantly less sensitive and specific than FNAB for a cytogenetic prognostication [8,33].

#### 2.4.2. Vitrectomy-Based Biopsy

The transvitreal (indirect) approach includes the following sampling techniques: vitrectomy-assisted biopsy, Essen forceps biopsy and incisional biopsy.

The transvitreal retinochoroidal (TVRC) biopsy, performed with a 25-G or 27-G vitreous cutter, usually yields adequate specimens for cytogenetic and cytopathologic examination [20]. 27-G is preferred over other larger gauges since it produces a smaller retinotomy and can be used to biopsy thinner lesions [34]. The procedure involves a three-port pars plana setup, even if some surgeons avoid infusion. The vitreous cutter is advanced without cutting through the vitreous cavity and retina into the choroidal tumor under a direct transpupillary visualization. Subsequently, a vitreous separation can be induced over the tumor, and a thorough vitrectomy can be performed over the intended biopsy site, to avoid vitreoretinal incarceration with the vitreous cutter during the biopsy procedure. The intraocular pressure is elevated to avoid bleeding [34]. The retina overlying the tumor may be incised by a sharp intraocular Sato knife just to allow the transretinal entrance of the 0.9 mm-thick vitreous cutter. The vitreous cutter is then inserted into the tumor with a high aspiration setting (400–600 mmHg) and a low cutting frequency (80–300/min) [26]. No cryotherapy or laser treatment is usually performed at the retinotomy site. The scleral incisions are sutured and cryotherapy performed [20,34]. The sample is obtained by continuous suction and cutting until sufficient material is observed in the tubing [35,36]. The seeding of the tumor cells during intraocular biopsy in UM remains a significant concern, but has been limited by refined techniques and smaller cannulas [20]. Still, to reduce the potential risk of tumor seeding, some centers advise that biopsy is performed after the completion of tumor radiation, as genetic prognostication seems to be unaffected shortly after irradiation, mainly because irradiation induces random lesions in the DNA and thus up to 6 months after irradiation there is no change in tumor specific genetics. [37]. However, other authors have suggested that radiations can alter genetic testing in UM and that the radiation effect might depend on the time interval between radiation and genetic testing [38].

Transvitreal biopsy using an Essen forceps is a suture-less procedure that requires a 23-G 3 port pars plana vitrectomy, followed by a 0.6 mm incision in the retina to allow the advancement of the open Essen forceps into the tumor [39]. The sample is grasped by the forceps and withdrawn through the vitreous cavity and scleral port. The procedure may provide for a larger tissue specimen but entails a theoretically potential risk of tumor seeding in samples larger than 0.6 mm, as they can get stuck at the entry site of the scleral port [39]. The main advantage of using Essen biopsy forceps is the large amount of tissue obtained by the procedure [26].

Pars plana vitrectomy-assisted incisional biopsies include a full 23-G/20-G 3 port pars plana vitrectomy [40]. A 20 G diamond knife is used to make a retinal incision, followed by the excision of a 1 mm^3^ tissue sample. The tumor sample is subsequently removed through the sclerotomies by end-gripping forceps. Retinal diathermy is used to minimize bleeding. This procedure allows for large samples, adequate for histopathological assessment, but entails a significant risk of retinal detachment [40]. The main biopsy approaches for posterior ocular tumors are summarized in Table 1.

#### 2.4.3. Safety

Several potential risks may be associated with intraocular tumor biopsy, including hemorrhages, retinal detachment, cataract and endophthalmitis, as well as tumor seeding or extraocular spread. The main potential risk in performing a biopsy in UM is tumor dissemination. The mechanical disruption of intratumor blood vessels during the biopsy procedure carries a theoretical risk of an intravascular tumor cells spread [41]. Nonetheless, it is well known that tumor cells’ metastatic potential is not related to mechanical dissemination, but to biological tumor characteristics [42]. However, local dissemination can theoretically occur by tumor cells being passively dragged along the needle shaft into the vitreous body and scleral wall or by the active migration of tumor cells through the lesion made by the biopsy needle [43]. It is of great importance to minimize seeding risk, as local recurrence carries an increased risk of metastatic disease [44]. The minimally invasive TVRC biopsy is characterized by several features that can theoretically decrease the local seeding risk. The intact vitreous body decreases the flow inside the eye and thereby potentially reduces the intraocular spread of tumor cells. A histopathological examination of needle tracts revealed a lower number of seeded tumor cells following transvitreal biopsies compared to trans-scleral biopsies [43,45]. However, because of the location of the transscleral biopsy site usually within the field of radiation, differently from the transvitreal entry site [26], the transscleral approach at the time of the brachytherapy appears to be the safer approach. One case of seeding inside the vitreous body following a transvitreal biopsy has been described in a clinical study, but the clinical significance of seeded tumor cells in the vitreous body is doubtful [45]. Indeed, the spontaneous migration of tumor cells into the vitreous body in treatment naive patient eyes does not seem to be associated with a bad prognosis [46]. The access through pars plana allows for the surveillance of spillover of tumor cells and late local recurrence at the biopsy entry point. Furthermore, the scleral ports likely reduce the risk of tumor seeding in the sclera [27]. However, the risk of scleral seeding is not entirely eliminated, as demonstrated by case reports of extraocular recurrence at the scleral port entry site following a transvitreal biopsy [47,48]. The vitrector system allows for sufficient biopsy material with a single pass compared to transscleral biopsies, which usually demand several passes, increasing the number of seeded tumor cells mainly if multiple needle tracts are performed [41]. Nevertheless, FNAB of UM is generally accepted to be a safe procedure [4,27,49,50]. In one prospective case series, Singh et al. described outcomes in 150 eyes which had FNAB of UM, including 71 eyes which had a partial thickness scleral flap at the time of their transscleral biopsy and plaque placement [27]. They reported no tumor recurrence, at 37 months of follow-up [27]. However, a few rare case of extrascleral tumor extension after FNAB have been reported in the literature [47,51,52,53]. Different precautions have been suggested to limit this occurrence, including using a small gauge instrument (25- or 27-G), performing a peritomy over the biopsy site, maintaining a dry field during biopsy with minimal infuse use, releasing negative pressure before withdrawing the needle, using a transscleral cannula to create a protected needle tract, and applying cryotherapy at the sclerotomies [7,26,54]. Moreover the subsequent application of radiation therapy may further reduce the risk, sterilizing the seeded tumor cells inside the eye, even if the delayed sampling, at a long interval after radiotherapy, may affect the genetic testing results [11,37,38,55,56]. Siegel et al. evaluated three eyes with UM that had FNAB, using a lamellar scleral flap at the time of the plaque brachytherapy placement and subsequently developed scleral thinning over the flap site [57]. Two eyes then developed melanocytic proliferation over the site of the scleral flap. The third patient exhibited scleral thinning and evidence of tumor growth on ultrasound, with extraocular tumor extension confirmed at histopathology. The authors concluded that patients with scleral flaps created for the biopsy of UM are at risk of scleral thinning and extrascleral extension of tumor recurrence through the flap. Given the potential to confuse the clinical picture, the authors recommend to take precautions when using a lamellar scleral flap during transscleral biopsy [57]. Shields et al. evaluated the safety of FNAB in 140 patients. Complications were minimal with no case of extrascleral tumor extensions [58].

More recently, Bagger et al. followed 1637 patients with UM for a total of 3.9 and 8.4 thousand person-years of observation for transvitreally biopsied (TVRC, FNAB or Essen forceps biopsy) and non-biopsied patients, respectively [6]. They found no significant increase in the all-cause mortality and melanoma-specific mortality among biopsied patients compared to non-biopsied patients [6]. These findings are in accordance with previous case series of intraocular biopsy in UM, where no excess mortality has been reported [24]. In the population study of Bagger et al., eighty-two patients (96.5%) presented with vitreous hemorrhage on the first day after surgery [6]. In 71 of the patients (86.6%), the vitreous hemorrhage cleared spontaneously within 2 years. Five patients (5.9%) underwent vitrectomy due to a persistent vitreous hemorrhage [6]. Similar frequencies have been reported by other groups using vitreous cutter biopsies, whereas transvitreal FNAB seems to cause lower rates of vitreous hemorrhages [3,20,26,31,35,36,59]. Even lower risks are due to FNAB obtained through the transscleral approach [50]. In general, FNAB seems to be safer than transvitreal biopsy in terms of vitreous hemorrhages, probably because of a limited manipulation of the tumor tissue and vessels [6,26]. It has been proposed that performing a full vitrectomy during biopsy can entail a faster resolution of vitreous hemorrhage [30]. A rarer complication of ocular tumor biopsy is rhegmatogenous retinal detachment, with a slightly higher incidence following TVRC than after transvitreal FNAB [4,31,36,59]. Even in cases with exudative retinal detachment already present at the time of sampling, the procedure does not seem to worsen the detachment [6,26]. Associated retinal tears tend to seal spontaneously, maybe because of the buckle effect of the tumor mass. In some cases, laser treatment may be necessary [6,20,26]. No significant cases of endophthalmitis following a tumor biopsy have been reported, probably due to the sterilizing action of radiations [60].

### 2.5. Biopsy for Cytogenetic Analysis

In a scenario where new drugs for systemic treatment develop continuously, it is mandatory to identify subgroups of patients amenable to receive a “tailored” optimal treatment [5,11,61]. Moreover, systemic adjuvant therapies may be theoretically more effective in treating microscopic rather than macroscopic tumor metastases, where multiple mechanisms of resistance usually develop. Tumor genetic and molecular factors may become appropriate targets for individualized therapies and a stratified enrolment in clinical trials. Actually, the more significant achievements have been reached in terms of prognostication, since chromosomic alterations in UM have proved to be highly predictive of metastatic risk [6,62]. An accurate prognostication allows for an individualized follow-up and systemic surveillance, effective patient counseling and the optimization of healthcare resources. In this context, prognostic biopsies assume a different meaning from histo- or cytologic biopsies, requiring a tailored approach.

**Figure 7:**
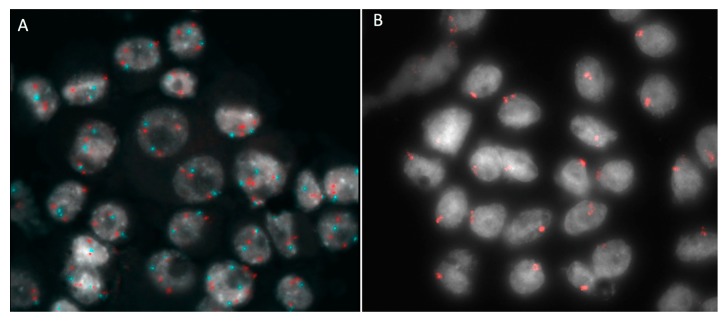
Fluorescence in situ hybridization analysis (FISH) in a case of posterior uveal melanoma sampled by fine needle aspiration biopsy. (**A**) FISH with locus specific probe for MYC gene (red) and for the centromere of chromosome 8 (light blue) confirmed the gain of 8q24 showing three copies of MYC gene in each cell; (**B**) The same case was also characterized by monosomy 3: cells with a single red hybridization signal have lost one chromosome 3.

The failure of FNAB to yield a sufficiently cellular specimen from a presumed choroidal or ciliary body melanoma for cytopathologic classification is a problem encountered in many reported series [2,5,11,20,29]. The principal factors associated with such an insufficiency are a limited tumor thickness, the differential diagnostic subcategory of the tumor (i.e., “unequivocal melanoma” versus “atypical but probable melanoma” versus “nevus versus melanoma”), and the intention category of the biopsy (i.e., diagnostic, investigational, prognostic) [31,63]. If the vitrectomy assisted biopsy has proved to provide sufficient material for both histopathological and genetic testing, this is not always achieved with FNAB, which allows for a similar percentage of sufficient sampling only when genetic tests are considered [6,7,27,64]. Traditional staging methods that use clinical and histologic prognostic factors, such as the American Joint Committee on Cancer (AJCC) tumor, node, metastasis (TNM) system, are used to stratify patients into general risk categories, but they do not provide a sufficient predictive accuracy to be used alone for patient care [65]. In 1996, Prescher et al. discovered a strong association of metastatic death with the loss of one chromosome 3 (monosomy 3) in a primary tumor [66] (Figure 3; Figure 7B).

**Figure 8:**
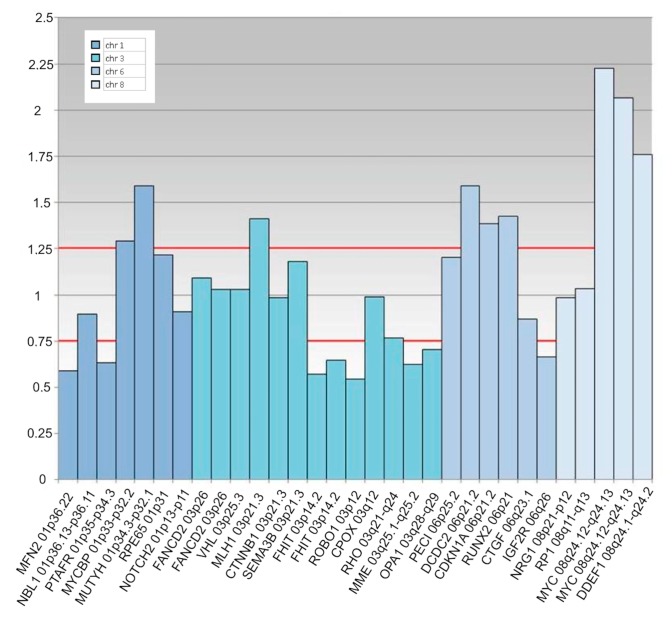
Multiplex Ligation Probe Amplification analysis in a case of posterior uveal melanoma sampled by fine needle aspiration biopsy. The tumor is characterized by losses on the chromosome 1p, 6q and all along the arm of chromosome 3, including the centromeric region until 3p14; gains were also present in the 6p and 8q regions, with an amplification of the MYC gene (8q24.12–8q24.13).

Later, other chromosomic alterations have proved to be significant in terms of prognosis, such as chromosome 8q gain (Figure 7A) and chromosome 1p loss, which correlated with an increased mortality, and chromosome 6 rearrangements have also been shown to be frequent in UM [64,67]. The consideration of these additional risk factors may improve the correlation with the metastatic disease [68]. In 2017, Shields et al. published a large cohort of 1059 samples, analyzing chromosomes 3, 6 and 8, and correlated genetic results with clinical features (phenotype—genotype correlation) [64]. With regards to tumor size, they found an association with single-chromosomal abnormalities (small/medium/large), in particular with the loss of disomy chromosome 3 (35%/52%/65%, respectively), loss of disomy 6 (15%/34%/51%), and loss of disomy 8 (19%/41%/69%), indicating that a greater tumor size was correlated with a greater single-chromosome mutational profile [64]. The evaluation of UM size, as an independent prognostic factor, seems to add predictive power to the genetic analysis for estimating the likelihood of metastasis [7,69,70]. Genetic alteration has increasingly been used as a marker for prognostic tests in patients with UM, and chromosomic and genetic tests have assumed a progressively more relevant role in the management of UM patients. Several analysis techniques have been developed, from karyotyping and fluorescence in situ hybridization (FISH) (Figure 2 and Figure 7), to multiplex ligation-dependent probe amplification (MLPA) (Figure 8), single nucleotide polymorphism (SNP) and gene expression profiling (GEP), which has been proposed to be less, but still affected by genetic tumor heterogeneity, compared to the other modalities [6,71,72].

**Figure 9:**
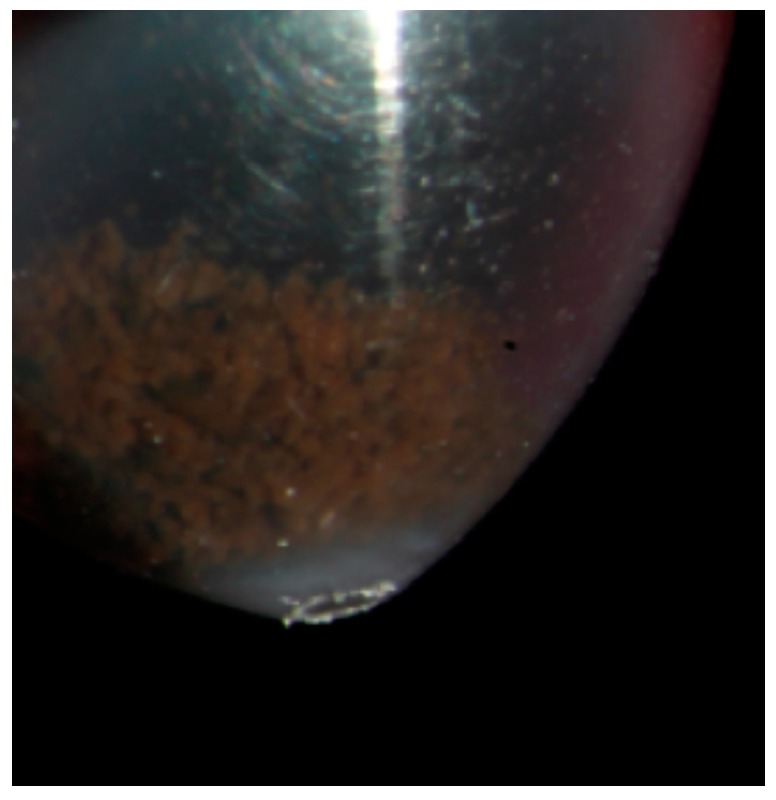
Fine needle aspiration biopsy sample in a medium sized posterior uveal melanoma: a large number of cells is obtained and collected in a vial with the culture medium Roswell Park Memorial Institute (RPMI) 1640 (Euroclone Life Science, Pero-MI, Italy) before the genetic analysis. The obtained material is visible in the vial as a brown deposit at the bottom of the tube.

UM heterogeneity is considered a consequence of cancer pathogenesis. Cancer development is often associated with genomic instability and the acquisition of genomic heterogeneity generating both clonal and nonclonal tumor cell populations. Morphologic heterogeneity is well recognized in UM, showing variable proportions of epithelioid and spindle cells [73]. Due to heterogeneity, diagnosis using FNAB is complex, given that most tumor samples are obtained by a single pass. This is particularly true when the base of the tumor is sampled, where heterogeneity has proved to be more significant [6,73]. Therefore, tumor heterogeneity may interfere with a correct prediction of the patient’s prognosis [6,73]. Some cases of eyes without a loss of chromosome 3 developing metastatic disease have been reported. It is possible that these tumors evolved in a different manner, but it may also be due to the inability to detect, for example, a partial loss of chromosome 3 (Figure 8), isodisomy 3 (duplication of one copy of a chromosome), or it may be due to intratumor heterogeneity [61]. Therefore, biopsy techniques providing for a large sample (Figure 9) may reduce the risk of tumor misclassification.

The chosen genetic analysis technique may have a role in the correct tumor characterization [6]. MLPA, which analyzes the gain or loss of chromosomal material using the DNA in tumor cells, has been proven to require smaller biopsy sample than FISH, and GEP seems to better reflect the tumor microenvironment than the genetic changes in the tumor cells [7,26,71].

Gene expression profiling is a transcriptional method of cellular analysis that takes a “snapshot” of the tumor microenvironment that can be used to predict the metastatic potential of the tumor, analyzing genes of interest. Because tumor sample requirements are generally lower for the GEP assay, it has a lower technical failure rate than chromosomal assays [6,26,71,74]. The prognostic value of a standardized 15-genes assay developed by Harbour and coworkers using GEP has been validated in a multi-center clinical trial [8]. Patients having a primary UM categorized by this standardized assay as GEP class 2 experienced a substantially higher rate of metastasis than patients whose tumor was categorized as GEP class 1 did. Tumors with monosomy 3 corresponded to class 2 tumors as classified by GEP, whereas tumors without a loss of chromosome 3 referred to class 1 tumors. Most of the tumor specimens evaluated in the multi-center validation trial were cellular aspirates obtained by FNAB at the time of or shortly prior to the initial tumor treatment. In almost all of these cases, a single tumor site was sampled by FNAB for GEP testing and classification and was assumed to be representative of the tumor as a whole [75]. One recently identified prognostic factor is the mutation of BRCA1-associated protein 1 (BAP1). BAP1 is involved in various biological processes, including the response to DNA damage, cell cycle regulation and cell growth [76]. The presence of inactivating somatic or germline BAP1 mutations, often in conjunction with chromosome 3 monosomy, the loss of BAP1 expression, or the lack of immunohistochemical staining, have all been associated with metastasizing UM [77,78]. Defining the germline vs. somatic nature of BAP1 mutations in UM may inform the individual about both the risk of metastasis, and the probable time to metastasis, which are critically important outcomes for the individual. This information can also change the cascade screening and surveillance of family members [79].

PRAME and EIF1AX genes have been also correlated to a higher and decreased risk of metastases, respectively, in UM, whereas GNAQ or GNA11 gene mutations are characteristics of melanocytic origin cells, also of nevi, thus not being particularly informative about prognosis but confirming the origin of the sampled tissue. This may be significant, since existing prognostic tests, including GEP, may also provide genetic results in cells not derived from melanoma, but from other tissues. Another gene involved in intermediate risk UM is SF3B1, whose mutations have been observed in late metastasizing tumors [7,67]. The identification of these genetic mutations require continuous efforts in developing sampling and analysis techniques [67].

A summary on main studies using biopsy for intraocular tumors, with the reported complications and purposes has been provided in Table 2.

### 2.6. Liquid Biopsy

Even if the genetic component has proved to have a primary role in the mechanisms of UM development, progression and metastasis, specific angiogenic, immunologic and inflammatory pathways have been also related to UM progression and spreading. In this context, the analysis of cytokines, chemokines and growth factors implicated in these mechanisms and individuated in ocular fluids, using minimally invasive, well known and safe aqueous and vitreous humor sampling, has allowed for a better characterization of the UM microenvironment. The detection of elevated levels of proinflammatory cytokines has confirmed the relevance of the recently characterized inflammatory phenotype of UM, defined as an increased number of T-lymphocytes and tumor-associated macrophages, and associated with the presence of high risk histological and genetic characteristics (epithelioid cells and monosomy 3) [80,81,82].

Furthermore, UM develops in an immunologically privileged environment, where both the adaptive and innate immune systems are suppressed. In addition, UM cells develop a series of mechanisms to escape immune surveillance. Therefore, immunotherapy has been recently proposed as a promising therapy, particularly in metastatic patients, also in view of the encouraging results obtained in cutaneous melanoma patients. The identification of immune-related factors may be involved in UM progression, and different aqueous humor concentrations may allow for an early detection of tumors with a greater propensity for diffusion, providing a possible new target for individualized therapies [83].

Recently, the detection and characterization of circulating tumor cells has also assumed growing relevance in the management of various cancers [84]. Even if no evidence of metastatic disease is clinically demonstrable at the time of the UM diagnosis in the majority of cases, micro-metastases have been suggested to form several years before clinically detectable metastases, even at the time of diagnosis [42]. This notion, together with the pure hematogenous dissemination of UM cells, would support the rationale of researching circulating melanoma cells (CMCs), considered as predecessors of a metastatic settlement, in UM patients [84]. CMC studies have proved that CMCs may be detected in almost all UM patients, confirming that other mechanisms are involved in the metastatic process. Therefore, the analysis of CMC genetic, immune and molecular alterations (and not only the mere presence) might be more informative than primary tumor analysis as regards metastatic potential [84]. These evidences suggest that “liquid biopsies”, also in relation to their safe nature, have a substantial potential to serve as an additional tool in the care of UM patients and in the better understanding of the pathological processes involved in UM spreading. However, it is not clear whether liquid biopsy will be able to replace tumor biopsy studies in the near future. Moreover, we recommend caution in the current use of this technique, mainly in relation to the absence of clear indications in UM patients.

## 3. Discussion

**Figure 10:**
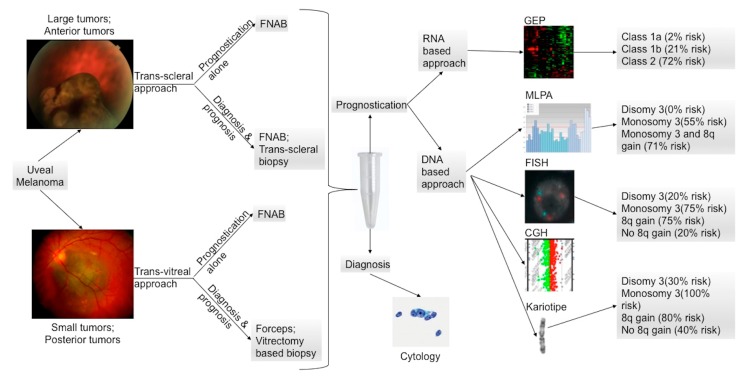
Posterior uveal melanoma prognostic test flow-chart modified from Schopper et al. [85]. Large and anterior tumors are commonly biopsied using a trans-scleral approach, whereas posterior tumors and small tumors are better reached by a transvitreal approach. When possible, FNAB (fine needle aspiration biopsy) should be used because it is considered to be the less invasive technique. Current prognostic tests rely on either DNA or RNA extraction from tumor specimens. FISH (fluorescence in situ hybridization), CGH (comparative genomic hybridization), MLPA (multiplex ligation-dependent probe amplification), and karyotyping are the most common used techniques for the DNA analysis. GEP (gene expression profiling) is the preferred technique for the RNA-based prognostication. An estimated 10-year metastasis-free survival is listed based on publications on the karyotype analysis [86], FISH [87], and MLPA [88]. An estimated 5-year metastasis-free survival based on GEP classification is also listed [89].

UM biopsy includes a variety of techniques with different targets and purposes, which have become more and more differentiated with the increase in knowledge about UM pathogenesis. In clinical practice, it may have both diagnostic and prognostic significance. However, with the progressive improvement in clinical diagnostic non-invasive techniques, biopsy for diagnostic purposes is reserved for selected cases in which the confirmation of a diagnosis could modify the subsequent management. Conversely, following the constant and continuous improvement of systemic therapies, biopsy is gaining more importance as a means of genetic analysis to set up therapies aimed at the specific case, based also on the stratification of the clinical risk (Figure 10).

The availability of accurate and validated prognostic information can have an impact on the selection of a management or treatment plan, including surveillance, reporting and initiation of therapy within the clinic or the experimental clinical environment corresponding to the metastatic risk. Personalized therapy based on specific oncogenic targets in the individual tumor requires tissue for risk assessment and genetic profiling to select and match patients to the most effective treatment. Unfortunately, molecular prognostic tests still have little impact on treatment decisions among ophthalmologists who diagnose and treat patients with UM.

However, the prognostic significance of UM biopsy has achieved a high precision, becoming the actual standard approach in counseling UM patients. When appropriately performed, tumor biopsy can be considered a safe procedure that can be modulated, using a variety of surgical techniques, depending on the size and location of the tumor and each eye characteristics. The main limitations include tumor heterogeneity, which remains a significant cause of misclassification, even with the most recent analysis technologies, and limited but still present risks related to the invasiveness of the procedure. Further research is needed to facilitate the development of new effective targeted treatments, which can improve the stagnated survival in patients with UM. Thus, the continuous development and refinement of tumor sampling in UM are warranted, and new techniques for minimally invasive biopsy techniques are alluring.

## 4. Materials and Methods

To identify potentially relevant articles in the medical literature, we searched MEDLINE^®^ (8600 Rocksville Pike, Bethesda, MD 20894, USA) for English language articles published in the last 20 years. MEDLINE^®^ was queried using the following search terms (used both alone and in combination for advanced research): ocular tumor biopsy, ocular tumor fine needle aspiration biopsy, transvitreal tumor biopsy, transscleral tumor biopsy, ocular cytogenetic, and uveal melanoma molecular prognosis. Additional articles were identified by reviewing the references of examined publications. To identify potentially relevant articles to include in this review, two investigators reviewed each paper, and the most significant were included. Review and study articles were preferred to case reports or case series. Articles included in the reference list were fully examined by the authors.

## 5. Conclusions

Tumor sampling procedures are commonly performed not to confirm the diagnosis of UM, but to obtain a tissue sample for prognostication, which can help assess the patient-specific metastatic risk. The obtained genetic information can also influence the surveillance timing and metastatic screening type of patients. In spite of the widespread use of biopsies in general surgical practice, in ophthalmic oncology the indications and contraindications for tumor biopsy continue to be under debate.

## Figures and Tables

**Table 1:** Biopsy approach techniques.

Surgical Approach	Biopsy Type	Gauge Needle	Main Advantages	Main Disadvantages	Sample Use
Transscleral	Excisional biopsy	NA	Large sample	Risk of seeding when performed before irradiation Surgically demanding Ocular complications	histopathology and genetics
	Fine-Needle Aspiration biopsy	25 G–30 G	Cheap; Simple technique	Relatively small amount of tissue Not indicated in small tumors Not indicated in post-equatorial tumors	cytopathology and genetics
Transvitreal	Fine-Needle Aspiration biopsy	25 G–27 G	Cheap	Relatively small amount of tissue Risk of retinal complication and vitreous hemorrhages	cytopathology and genetics
	Vitrectomy-based biopsy	25 G–27 G	Relatively large amount of tissue	Expensive Risk of retinal complication and vitreous hemorrhages Requires vitrectomy expertise	cytopathology and genetics
	Essen forceps biopsy	23 G	Relatively large amount of tissue	Risk of seeding? Risk of retinal complication and vitreous hemorrhages	cytopathology and genetics
	Incisional biopsy	NA	Large sample	High risk of retinal complication and vitreous hemorrhages	histopathology and genetics
	Vitrectomy-based endoresection	25 G–27 G	Large sample	High risk of retinal complication and vitreous hemorrhages Surgically demanding Risk of seeding if performed before irradiation	histopathology and genetics

NA = Not applicable; G = Gauge; Table modified from Bagger MM et al. *Acta Ophthalmol*. **2018**, *96* Suppl A112, 1–28.

**Table 2:** Summary of main studies using biopsy for intraocular tumors.

Study	Type of Biopsy	Gauge	N. of Eyes	Tumour Location	Tumor Basal Diameter (Median, mm)	Tumour Thickness (Median, mm)	Adequacy	Complications	Purpose	Genetic Analysis
Woog et al., 1984 [18]	Aqueous tap	30	1	Iris	NA	NA	100%	None	Diag	NA
Glasgow et al., 1988 [41]	Various types	30	11	CH/CB	NA	NA	95%	Tumor cells in FNAB tract: direct 67%, indirect in 53%		NA
Char et al., 1995 [43]	Transscleral, Transvitreal FNAB	25	100	CH/CB	12.2	5.8	86%	Transient vitreous haemorrhage	Diag	NA
Eide et al., 1999 [29]	Various types	25	80	Iris, CH/CB	NA	NA	94%	Small haemorrhages (10%), retinal detachment (5%), traumatic cataract (1%)	Diag	NA
Cohen et al., 2001 [31]	Transvitreal FNAB	25	83	CH/CB	NA	5.3	88%	Small haemorrhage at the biopsy site (100%), vitreous haemorrhage (24%), endophthalmitis (1%)	Diag	NA
Augsburger et al., 2002 [2]	Transvitreal FNAB	25	34	CH/CB	8.0	2.4	65%	NA	Diag	NA
Bechrakis et al., 2002 [20]	Vitreous cutter	20	34	Iris, CH/CB	NA	NA	100% for iris 97% for choroid	Vitreous haemorrhage (6%), intraocular tumor spread (3%)	Diag	NA
Carminal et al., 2006 [53]	Transscleral FNAB	25	1	CH/CB	17	6.2	100%	Vitreous haemorrhage	Diag	NA
Char et al., 2006 [17]	Aqueous tap	25	22	Iris	NA	NA	69%	NA	Diag	NA
Midena et al., 2006 [11]	Transscleral FNAB	25	8	CH/CB	10.6	8.2	87.5%	None	Prog	FISH
Sen et al., 2006 [25]	Transscleral biopsy	25	14	CH/CB	NA	NA	93%	None	Diag/Prog	Cytogenetic
Shields et al., 2006 [19]	Iris FNAB	Various needles	100	Iris	9.0	2.5	99%	Partial hyphema (34%)	Diag	NA
Shields et al., 2007 [3]	Transvitreal (43%) and Transscleral (57%) FNAB	Various needles	56	CH/CB	9.7	2.7	67–97%	Transient vitreous haemorrhage (55%)	Prog	DNA amplification and MSA
Shields et al., 2007 [58]	Transvitreal (75%), transcleral (25%) FNAB	Various needles	140	Iris, CH/CB	9.7	3.9	97%	Local haemorrhage	Prog	DNA amplification and MSA
Bonaldi et al., 2008 [61]	Transcleral FNAB	NA	28	CH/CB	12.2	8.2	100%	None	Prog	FISH
Onken et al., 2010 [71]	Unspecified FNAB	25	609	CH/CB	NA	NA	100%	NA	Prog	GEP
Akgul et al., 2011 [39]	Transvitreal Essen forceps biopsy	23	20	CH/CB	NA	3.4	95%	Temporary punctual bleeding (15%)	Diag	NA
Petousis et al., 2011 [21]	Biopsy using vitreous cutter	25	55	Iris	5.2	1.8	96–100%	Increased intraocular pressure (11%), hyphema (2%), flare (2%), persistent pupillary defect (2%)	Diag	NA
Raja et al., 2011 [47]	Vitrectomy-based biopsy	25	1	CH/CB	17.8	4.6	100%	Extraocular seeding at 14 months of follow-up	Diag	Cytogenetic
Shields et al., 2011 [49]	Transscleral and Transvitreal FNAB	27	500	Iris, CH/CB	10	3.8	100%	None	Prog	DNA amplification and MSA
Ewens et al., 2012 [9]	FNAB compared with post-enucleation biopsy	NA	320	Iris, CH/CB	12	4.5	100%	NA	Prog	Whole genome array-based assay
McCannel et al., 2012 [24]	Transscleral FNAB	30	170	CH/CB	10.8	4.8	53–91%	None	Prog	FISH
Onken et al., 2012 [8]	Unspecified FNAB, post-enucleation FNAB, tumor resection	NA	459	CH/CB	10.8	6.3	78%	NA	Prog	GEP
Abi-Ayad et al., 2013 [35]	Vitrectomy-based biopsy	25	9	CH/CB	12.9	7.2	100%	Blood clot at the biopsy site (89%), minimal vitreous haemorrhage (89%)	Diag	NA
Augsburger et al., 2013 [32]	Unspecified FNAB	NA	302	CH/CB	NA	NA	86%	NA	Diag/Prog	NA
Schefler et al., 2013 [52]	Various types	NA	4	CH/CB	NA	NA	100%	Extraocular extension during follow-up	Diag	NA
Seregard et al., 2013 [40]	Vitrectomy-based biopsy	23	43	CH/CB	NA	4.0	95%	Progression of pre-existing retinal detachment (12%), transient increase of IOP > 40 mm Hg (14%)	Diag	NA
Correa et al., 2014 [33]	Transvitreal FNAB	25	159	CH/CB	NA	NA	88–99%	NA	Diag/Prog	GEP
Gold et al., 2014 [56]	Unspecified FNAB	NA	3	CH/CB	13.7	4.1	100%	NA	Prog	GEP
Grixti et al., 2014 [60]	Transvitreal and transcleral FNAB	25	739	NA	NA	NA	NA	Persistent vitreous hemorrhage (2%), rhegmatogenous retinal detachment (0.7%), endophthalmitis (0.14%)	Diag/Prog	NA
Augsburger et al., 2015 [72]	Unspecified FNAB	25	80	CH/CB	12.3	5.8	98%	NA	Prog	GEP
Coupland et al., 2015 [55]	Various types	25	28	CH/CB	15	6.9	50%	NA	Diag/Prog	MLPA; MSA
Correa et al., 2016 [70]	Unspecified FNAB	NA	299	CH/CB	NA	NA	100%	NA	Prog	GEP
Hussain et al., 2016 [37]	Vitrectomy-based biopsy	25	102	CH/CB	12	3.5	100%	NA	Diag/Prog	GEP
Mashayekhi et al., 2016 [51]	Transscleral FNAB	27	1	CH/CB	16	10.2	100%	Extraocular extension at 18 months follow-up	Prog	Cytogenetic
Sellam et al., 2016 [50]	Transscleral, Transvitreal FNAB	Various needles	217	CH/CB	13.9	8.4	77.9%	Vitreal haemorrhage (14%)	Prog	Array CGH
Singh et al., 2016 [27]	Various types	25	150	Iris, CH/CB	NA	NA	92%	Persistent haemorrhage (subretinal haemorrhage or vitreous) (1%) and rhegmatogenous retinal detachment (1%)	Diag/Prog	FISH
Angi et al., 2017 [28]	various types	25	232	CH/CB	11.4	3.4	95%	Transient localised bleeding (8%), vitreous haemorrhage (8%), retinal detachment (1%) and retinal perforation (1%)	Prog	MLPA; MSA
Finger et al., 2017 [23]	Surgical biopsy	25	7	Iris	NA	NA	100%	None	Diag	NA
Grewal et al., 2017 [36]	Vitrectomy-based biopsy	27	18	CH/CB	8.6	3.3	89%	Vitreous haemorrhage (72%), rhegmatogenous RD (11%)	Diag/Prog	GEP
Kim et al., 2017 [45]	Transvitreal FNAB	25, 27	10	CH/CB	15.7	8.7	100%	NA	Prog	GEP
Koch et al., 2017 [48]	Vitrectomy-based biopsy	25	1	CH/CB	NA	NA	100%	Extraocular seeding at 3.5 years of follow-up	Diag	NA
Nagiel et al., 2017 [59]	Vitrectomy-based biopsy	27	17	CH/CB	9.4	1.7	100%	Focal vitreous haemorrhage (76%), diffuse vitreous haemorrhage (6%)	Prog	GEP, MLPA
Reddy et al., 2017 [30]	Transvitreal FNAB	25	57	CH/CB	13.1	5.0	100%	Transient vitreous haemorrhage (2%)	Prog	GEP
Shields et al., 2017 [62]	Unspecified FNAB	NA	1059	Iris, CH/CB	11	5	96%	NA	Prog	Whole genome array-based assay
Singh et al., 2017 [54]	Transvitreal FNAB	25	20	CH/CB	NA	NA	80%	Vitreous haemorrhage (5%)	Diag/Prog	MLPA
Siegel et al., 2018 [57]	Transscleral FNAB	Various needles	3	CH/CB	NA	NA	NA	Scleral thinning at follow-up	Prog	GEP
Tang et al., 2018 [34]	Vitrectomy-based biopsy	27	1	CH/CB	11.0	4.0	100%	NA	Diag/Prog	GEP

MLPA = multiplex-ligation probe amplification; MSA = microsatellite assay; GEP = gene expression profiling; CGH = comparative genomic hybridization analysis; CH/CB = choroid and ciliary body; NA = not applicable; IOP = intraocular pressure; FNAB = Fine needle aspiration biopsy; Diag = Diagnostic; Prog = Prognostication; Diag/Prog = diagnostic and prognostic.
